# Patient and researcher perspectives on facilitating patient and public involvement in rheumatology research

**DOI:** 10.1002/msc.1171

**Published:** 2016-12-16

**Authors:** Judith Pollock, Karim Raza, Arthur G. Pratt, Helen Hanson, Stefan Siebert, Andrew Filer, John D. Isaacs, Christopher D. Buckley, Iain B. McInnes, Marie Falahee

**Affiliations:** ^1^ Institute of Infection, Immunity and Inflammation University of Glasgow Glasgow UK; ^2^ Institute of Inflammation and Ageing, College of Medical and Dental Sciences University of Birmingham Birmingham UK; ^3^ Sandwell and West Birmingham Hospitals NHS Trust Birmingham UK; ^4^ National Institute for Health Research Newcastle Biomedical Research Centre Newcastle upon Tyne Hospitals NHS Foundation Trust and Newcastle University Newcastle upon Tyne UK; ^5^ University Hospitals Birmingham NHS Foundation Trust Birmingham UK

**Keywords:** Patient and public involvement, rheumatology research, rheumatoid arthritis, conference report, stakeholder perspectives

## 1 INTRODUCTION

The Arthritis Research UK Rheumatoid Arthritis Pathogenesis Centre of Excellence (RACE) is a partnership between researchers at Newcastle University, the University of Birmingham and the University of Glasgow. Established in 2013, it is funded for 5 years, with a grant of £2.5 million from Arthritis Research UK and a further £4 million pledged by the three universities.

Rheumatoid arthritis (RA) is a chronic inflammatory autoimmune condition that affects the joints and internal organs. Synovial inflammation can cause cartilage and bone damage with resultant joint destruction and associated disability. RACE aims to identify biological mechanisms involved in the initiation of RA and its progression to a chronic disease, and to identify novel therapeutic targets for control and cure.

It has long been recognized that patients' experience complements researchers' skills (Hewlett et al., 2006). At the outset of the RACE project, it was agreed that patient engagement and involvement was crucial to facilitate the translation of research undertaken by the centre into public benefit. The present paper reports on a conference held at the University of Birmingham in January 2016 to bring together patient and public involvement (PPI) representatives from each hub, and to provide patients' and carers' perspectives on the following questions:

- What should research charities focus their spending on?
- How can people with RA influence the research agenda?
- What are the best ways for scientists to feed back to patients, relatives and carers about research findings?

## 2 BACKGROUND

PPI in all stages of the research process is advocated by funding bodies and policy makers as a way of enhancing the relevance, quality and efficiency of research (INVOLVE, 2012; National Institute for Health Research, 2015). The input of PPI partners has been shown to enhance the design of clinical trials (Brett et al., 2014), development of patient‐relevant research ideas (Bergsten et al., 2014), agenda setting (De Wit, Abma, Koelewijn‐van Loon, Collins, & Kirwan, 2013) and developing appropriate patient‐reported outcomes for clinical trials (de Wit, Kvien, & Gossec, 2015). Evidence suggests that patients identify different treatment priorities to clinicians (da Silva et al., 2010; Kwoh & Ibrahim, 2001). This reinforces the case for involving patients in the design of research to ensure that their priorities are addressed, and to improve the quality and impact of that research.

Patients have been involved in rheumatology research at each of the three hubs prior to the inception of RACE; therefore, the project offers a unique opportunity to bring together patient partners from each of the hubs, to share experiences and best practice. Details of these groups can be found in Table 1.

**Table 1:** Patient involvement in rheumatology research

Birmingham	The Birmingham Rheumatology Research Patient Partnership (R2P2; http://www.bham.ac.uk/r2p2) is an established partnership between people with rheumatoid arthritis and/or Sjögren's syndrome and rheumatology researchers at the University of Birmingham, Sandwell, and West Birmingham Hospitals NHS Trust and University Hospitals Birmingham NHS Foundation Trust. It was officially launched in October 2014, building on the success of patient partner panels that had been established to support individual research projects. Members of R2P2 are actively involved in all aspects of the research process, including the development of grant applications; the design of study procedures and of participant‐facing research materials; the development of informational resources; and the dissemination of research findings via patient networks and support groups. Patient partners are co‐authors on papers, including those related to the development of international research recommendations (Gerlag et al., 2012); the development of patient questionnaires (Stack et al., 2015) and patient‐initiated research to determine why people with symptoms of rheumatoid arthritis delay seeking help (Tiwana, Rowland, Fincher, Raza, & Stack, 2015).
Glasgow	The Glasgow Patient Involvement in Rheumatology Research (PIRR) group is in the early stages of its development with patient representation on clinical trial steering groups and further recruitment of patient research partners under way. Identification of possible patient partners has been through the National Rheumatoid Arthritis Society (NRAS), Arthritis Care and clinicians. There has been informal patient involvement for a number of years, while research is a standing item on the agenda of the Patient Engagement sub‐group (which includes patient representatives) of the Rheumatology Managed Clinical Network of NHS Greater Glasgow and Clyde.
Newcastle upon Tyne	The Newcastle Public Involvement in Musculoskeletal Services (PIMS) Group held its first formal meeting in March 2015. This joint project between Newcastle University and the Newcastle upon Tyne Hospitals NHS Foundation Trust has two main objectives: To involve service users and carers in improving the quality of services and making services more responsive to the needs of the individuals who use them To involve service users and carers in different aspects of research projects at Newcastle upon Tyne Hospitals NHS Foundation Trust, providing a forum for researchers and the public/patient community to formally engage with each other Researchers also discuss future projects and feed back results to existing patient groups, including the North East Sjögren's Syndrome Association (NESSA) and the local NRAS group.

## 3 RACE PATIENT CONFERENCE

The conference was attended by two patient research partners from Birmingham, five from Glasgow and six from Newcastle, 11 of whom completed a questionnaire prior to the conference, to describe their existing experience of research involvement. The participants' previous involvement in research activities is summarized in Tables 2 and 3. Patient partners who attended the conference had limited previous research involvement but were enthusiastic to become involved, as evidenced by their attendance and comments at the conference.

**Table 2:** Frequency of patient research partners' previous experience of involvement in research projects

Activity	Never	1 research project	2–5 research projects	More than 5 research projects
**Developing grant application**	9	1	1	0
**Offering advice as a member of a project steering group**	9	0	1	1
**Developing research materials (e.g. patient information sheets)**	9	0	2	0
**Writing of research reports**	10	0	1	0

**Table 3:** Frequency of patient research partners' previous experience of involvement in research events

Events	Never	1 event	2–5 events	More than 5 events
**Setting research priorities**	10	1	0	0
**Sharing research findings with the general public**	9	0	2	0

Respondents were asked to describe any benefits they had experienced or anticipated from involvement in research. Similar responses were obtained from those who had previous experience of PPI and those who had none. These can be summarized as:

- A better understanding of current research
- An increased knowledge of the disease and how to alleviate symptoms
- An opportunity to support research and ensure that it is targeted at patient needs
- Improved dissemination among patient groups
- Access to the latest research

Respondents were also asked to describe any difficulties they had experienced or anticipated from PPI in research. Many had encountered or anticipated no difficulties, although some raised concerns about the time commitment required, the use of technical language or the possibility that deterioration in their health might result in them withdrawing or reducing their involvement.

Clinicians and researchers from each hub were also present, along with the RACE project manager and a representative from Arthritis Research UK. Following an introductory presentation on PPI and the existing contribution of patient partners to rheumatology research at each hub, delegates split into groups to facilitate discussion and debate of the main topic areas. A third group of scientific researchers and clinicians affiliated with the University of Birmingham met separately to discuss the same questions. The three groups were then brought together to share key discussion points, which are summarized below, under each topic.

## 4 OUTCOMES FROM GROUP DISCUSSIONS

### 4.1 What should research charities focus their spending on?

There were some key differences between the views expressed by patient representatives and the expectations of researchers and clinicians. The latter group felt that patients would wish resources to be directed towards development of new treatments, particularly for those with longstanding, difficult‐to‐treat RA, who are increasingly described as becoming a neglected group, in terms of research priorities.

However, patient representatives were of the opinion that research to identify and evaluate new treatments for RA would be driven by the pharmaceutical industry, and that charity funding should be focused on understanding the biological processes that cause disease and on predicting response to treatment. Other priorities for patients included:

- Evaluation of the effectiveness of self‐management strategies
- Development of treatments for RA‐related fatigue
- The development of educational and awareness‐raising initiatives about RA.

The researchers/clinicians anticipated that patients' priorities would feature research into the effectiveness of non‐pharmacological approaches for the management of RA. The patient group agreed that there was a need for evidence of the effectiveness of such approaches. Patient representatives also reported that a focus on the therapeutic effectiveness of treatments was more desirable than a focus on their cost‐effectiveness.

### 4.2 How can people with RA influence the research agenda?

The importance of PPI in research, and research agenda setting, was agreed by all three discussion groups. The challenges of involving patients in basic laboratory‐based research were also discussed, with particular reference to the language gap between patients and researchers. Examples of successful approaches to this problem in previous projects were shared, such as the development of a glossary for PPI partners taking part in the European Union's FP7 project “EuroTEAM” (Towards Early diagnosis and biomarker validation in Arthritis Management: www.team‐arthritis.eu). Many patient partners perceived opportunities to become involved in research to be limited, and reported a lack of public awareness of such opportunities, and of the benefits of becoming involved. Benefits that were suggested included the ability to access the latest research findings and to engage with researchers, as well as the opportunity to “give something back” for care received by contributing to the research process. It was suggested that access to such benefits should be publicized and routinely available to all patients.

A concern was raised by patients that those who become involved with research may not be representative of the wider patient group, and often develop extensive research expertise, leading to a possible loss of perspective. It was agreed that it would be important to identify ways to widen access to research involvement in order to mitigate this. Patient representatives commented that it is not always straightforward for patients to attend research meetings in person, and opportunities to learn about involvement and contribute to research in other ways (e.g. via email or Skype) were welcomed.

**Figure 1:**
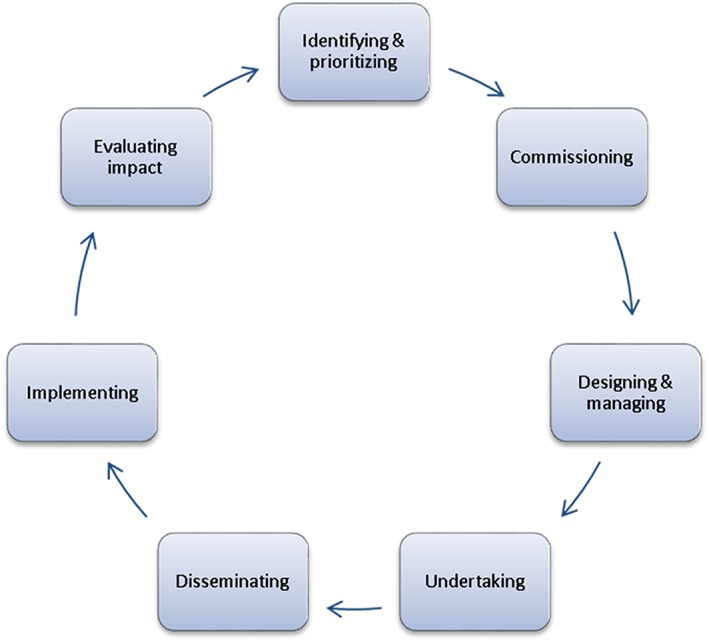
Ways in which people can be involved in the research cycle (INVOLVE, 2012)

The clinicians and researchers acknowledged the variety of opportunities for involving people throughout the research cycle (Figure 1), and highlighted the responsibility of researchers to be aware of the benefits of PPI, and to provide patients with comprehensive information about current research and future possibilities in order for them to be able to make fully informed suggestions and choices about research priorities. It was suggested that, in this way, the research cycle (Figure 1) should be seen as a virtuous cycle, where effective dissemination of research findings to patients facilitates the input of patients to the research agenda, and their ongoing engagement with the research process. This was echoed by patient representatives, who suggested that patient involvement should be an important aspect of training of researchers, including those involved in basic, laboratory‐based science.

### 4.3 What are the best ways for scientists to feed back to patients, relatives and carers about their research findings?

All groups agreed that patients could usefully be involved in the development and distribution of resources to communicate research findings. Furthermore, it was felt that groups such the Birmingham Rheumatology Research Patient Partnership (R2P2), the Glasgow Patient Involvement in Rheumatology Research (PIRR) group and the Newcastle Public Involvement in Musculoskeletal Services (PIMS) group, or other patient‐organized groups were an important mechanism for the dissemination of research findings.

It was suggested by patient representatives that it might be helpful to conceptualize three tiers of target audience: patients already actively engaged with research; patients who are not engaged; and the wider public. Thus, dissemination to local groups who can then cascade information via existing networks was perceived to be a useful approach. There was a strong consensus that this type of dissemination activity needed to occur at regular intervals, with a well‐publicized timetable.

Patient representatives involved in local groups suggested that they would value a database of local researchers who were willing to present their work to patients, along with opportunities to link with websites or mail‐outs associated with other local and national patient groups. While the importance of increased opportunities for social networking in this context was discussed, patient representatives felt that traditional media should not be neglected, as many patients do not use the internet or social media. Several patients suggested that patient‐friendly posters displayed in outpatient settings could be an effective route for local dissemination. In the researchers' group, there was discussion about whether clinicians or scientists were best placed to disseminate research findings to patients. Some concern was expressed by non‐clinical researchers that there would be a perception on the part of patients that all researchers would have clinical knowledge. However, several non‐clinical researchers described very positive experiences of face‐to‐face dissemination to patient groups, although it was acknowledged that preparatory work was necessary to ensure appropriate use of non‐technical language and management of patients' expectations in relation to the researcher's ability to answer clinical questions.

Patient representatives stressed the importance of communicating with patient partners throughout the research cycle, as well as on completion of specific research projects, and highlighted the need for training for researchers from an early stage of their career, to provide them with skills in the communication of research findings to patients and the wider public.

It was suggested that university press offices could be better engaged with scientists, and that there was a potentially useful role for patients to be involved in the identification of tractable items arising from research findings that might be seen as newsworthy.

## 5 CONCLUSIONS

In summary, there was wide agreement about the benefits of PPI in rheumatology research for all stakeholders. It was also generally agreed that research into the basic disease mechanisms of RA and prediction of disease outcome should be important priorities, although scientists/clinicians had expected patients to place greater emphasis on the development of new treatments and non‐pharmacological approaches. Participants shared their experiences of dissemination activities and identified effective strategies and opportunities for patient involvement in this context. An important role for patient groups and networks was identified in this context. We acknowledge that participating patients had previously expressed an interest in involvement with the RACE project, and their views may not represent those of all patients with RA. Further research is necessary to ascertain variations in patient perspectives in this context.

The RACE patient conference has identified key issues that should be addressed to facilitate patient involvement in rheumatology research:

- Increasing awareness of PPI and involvement opportunities
- Widening access to research involvement
- Increasing involvement of patients in research priority setting
- Training for researchers on the benefits of PPI and identification of involvement opportunities
- Training for researchers in the communication of research findings to patients
- Improving communication between researchers and patient groups
- Fostering robust networks for communication between researchers, patient groups, uninvolved patients and the wider public
- Increasing opportunities for sharing best practice in patient involvement and the dissemination of research findings to patients, carers, relatives and the wider public.
